# Preparation of a novel monoclonal antibody against active components of PHA-L from *Phaseolus vulgaris* and its functional characteristics

**DOI:** 10.1186/s12896-022-00761-7

**Published:** 2022-10-29

**Authors:** Peipei Wang, Junmei Hu, Jiaqi Duan, Shitong Min, Congliang Chen, Yue Zhu, Yan Pan, Yitian Wang, Dapeng Wei, Xia Wang

**Affiliations:** Department of Immunology, West China School of Basic Medical Sciences & Forensic Medicine, Sichuan University, 3-17 Renmin South Rd, Chengdu, 610041 Sichuan China

**Keywords:** PHA-L, Hybridoma clones, Monoclonal antibody, AICD

## Abstract

**Background:**

Leukocyte phytohemagglutinin (PHA-L), derived from the L4 tetramer of PHA, has been frequently employed as a mitogen to induce T lymphocyte proliferation in vitro. The biological application of PHA-L in cancer diagnosis and treatment has gained traction in recent years. However, it has been noted that PHA-L obtained using traditional procedures has a massive amount of impurities or toxic components, which interfere with the activity of PHA-L. Preparation of a monoclonal antibody against active PHA-L is a significant tool for studying PHA-L's function and therapeutic potential.

**Results:**

We successfully prepared monoclonal antibodies against the active components of PHA-L based on the whole PHA-L protein as an antigen, and found that monoclonal antibody 3C1C6G11 can be employed in western blot, immunofluorescence, and immunohistochemistry detection. Importantly, preliminary result shows that the mAb 3C1C6G11 may prevent PHA-L-induced cell aggregation and AICD (activation-induced cell death).

**Conclusions:**

The monoclonal antibody mAb 3C1C6G11 prepared in this study can be used as an effective tool for detecting PHA-L active components, investigating PHA-L's function and antineoplastic application.

**Supplementary Information:**

The online version contains supplementary material available at 10.1186/s12896-022-00761-7.

## Background

Plant lectins are non-immune carbohydrate-binding proteins, which exist widely in plant species. Since the mid-twentieth century, hundreds of plant lectins have been isolated and extensively studied for their biochemical properties, carbohydrate binding specificity, and biological activity [1, 2]. Lectins have been reported to have antifungal effects, inhibition of HIV reverse transcriptase, anti-proliferative and anti-tumor activities. It has also been proved to have broad prospects of biological and medical applications in microbial identification, blood typing, separation of glycoconjugates from cells, as a tool for differentiate malignant from benign tumors, and drug targeting in the gastrointestinal tract [3, 4]. It is therefore timely and significant to explore extensively the new biological effects and potential health benefits of lectins.

Phytohemagglutinin (PHA) is a high molecular glycoprotein composed of galactose, N-acetyl glucosamine and mannose, purified from red kidney bean (Phaseolus vulgaris). In general, PHA is a tetramer composed of two types of polypeptide chains called E and L, reflecting their preferential binding to erythrocytes and leukocytes, respectively. As a result, five potential tetrameric isomers (approximately 130 kD) can be formed randomly, namely E4, E3L1, E2L2, E1L3, and L4 [5, 6]. PHA-L, a four-subunit L4 tetramer, has been frequently employed as a mitogen to stimulate T lymphocyte proliferation in vitro. In recent years, the biomedical application of PHA-L in cancer diagnosis and treatment is gaining impetus. As aberrant glycosylation patterns in tumor cells have been shown to be associated with tumor progression in different tumor entities, PHA-L, which specially recognizes β (1,6)-branched oligosaccharides, has been applied to detecting the glycosylation status of tumor cells to provide information on metastasis and prognosis [7]. What's more, PHA-L has been reported to kill tumor cells through inducing apoptosis or autophagy both in cultured cells and in mice models, suggesting that PHA-L may serve as a potential antineoplastic drug in future cancer therapeutics [8].

Surprisingly, although plant lectins were first described several decades ago, many questions about their potential biological effects remain obscure, especially the toxicity of lectin from kidney bean (PHA) [9]. Thus, the toxicity and safety of PHA should be carefully evaluated when considering the application of it in therapeutics for humans. In our previous study, we have reported that PHA-L prepared from crude extracts of red kidney bean using standard strategies was a mixture of many ingredients that not only severely affected its function, but also produced toxic effects [10]. In recent years, the identification of the actual active proteins from crude extracts has gained tremendous emphasis, which may provide a new idea for further understanding of the function of PHA-L [11–13].

While PHA-L is involved in a variety of biological functions, particularly its biomedical application in cancer diagnosis and treatment, the lack of active PHA-L monoclonal antibody has greatly hindered PHA-L function research. Therefore, the development of active PHA-L monoclonal antibody is of great significance to thoroughly elucidate the potential antineoplastic application of PHA-L in tumor therapy.

In the present study, after immunization BALB/c mice using commercialized PHA-L, we first obtained four hybridoma clones secreting monoclonal antibodies that recognized intact PHA-L protein. Finally one of the hybridoma clones named mAb 3C1C6G11 was able to identify the active components of PHA-L that bound to human mononuclear cells. Immunoblotting assays showed that mAb 3C1C6G11 could strongly recognize the active components of PHA-L bound to cells. Immunohistochemistry and immunofluorescence assays showed that mAb 3C1C6G11 could also recognize the binding of PHA-L to any cells or PHA-L-treated mouse tumor tissue. Most importantly, mAb 3C1C6G11 blocked PHA-L-induced lymphocyte agglutination and PHA-L-mediated Jurkat cell AICD (activation-induced cell death).

Thus, the monoclonal antibody mAb 3C1C6G11 prepared in this study is not only suitable for western blot, immunofluorescence and immunohistochemistry assays, but also can be used to identify and purify functional PHA-L. Notably, mAb 3C1C6G11 blocks PHA-L induced cell agglutination and AICD, which may have therapeutic benefits in humans.

## Results

### Screening of positive hybridoma clones anti-active PHA-L and purification of the mAb 3C1C6G11

**Fig. 1:**
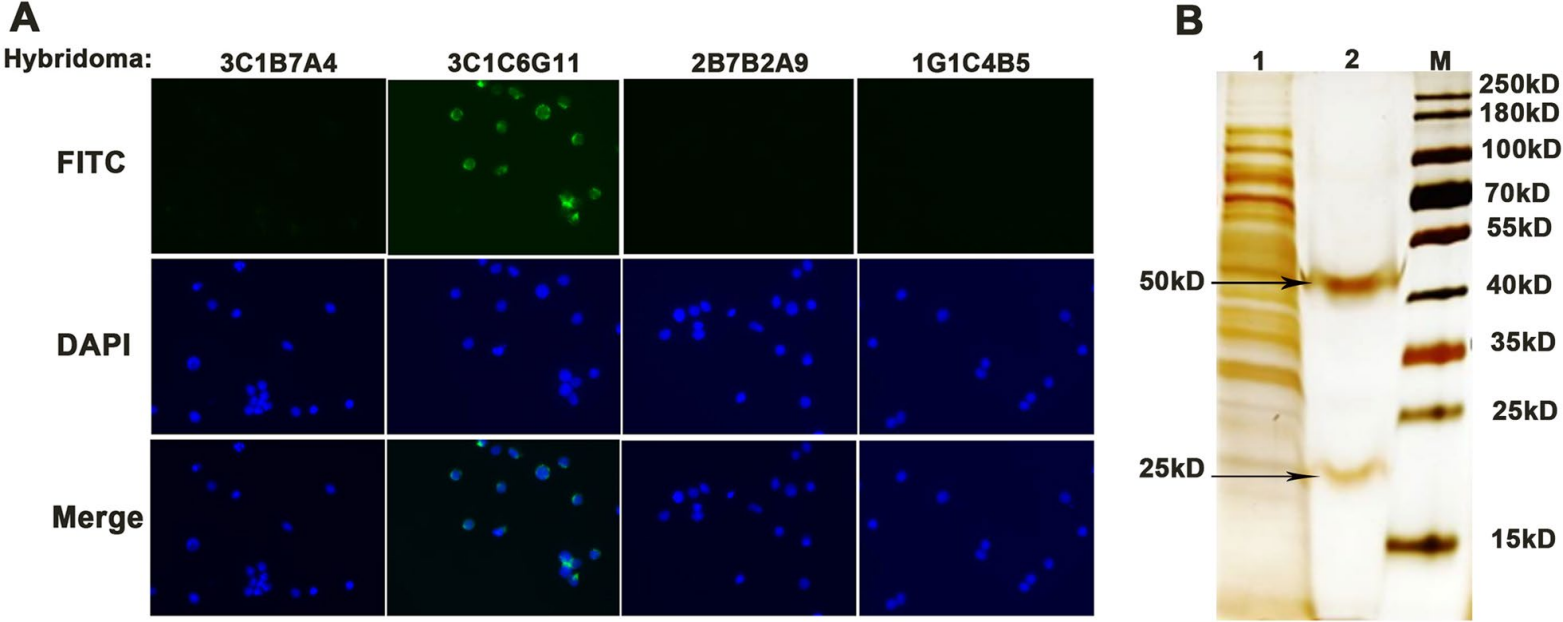
Screening and purification the mAb 3C1C6G11. **A** Representative picture of screening positive hybridoma cell lines against the active components of PHA-L by IF (original magnification × 200); **B** Silver staining analysis of purified mAb 3C1C6G11. M, Protein Ladder; Lane 1, unpurified ascites fluid after chromatography; Lane 2, purified ascites fluid. The positions of the target fragments are indicated by arrows

According to the general method for monoclonal antibody preparation, we obtained four hybridoma clones named 3C1B7A4、3C1C6G11、2B7B2A9 and 1G1C4B5, which secreted monoclonal antibodies that recognized intact PHA-L protein. Then, in order to screen monoclonal antibodies that specifically bind to PHA-L active ingredient, human mononuclear cells isolated from peripheral blood (PBMC) were used for identifying positive hybridoma clones with anti-PHA-L activity. As shown in Fig. 1A, when the supernatant of the 3C1C6G11 hybridoma cell culture was added in a 1:20 dilution, the PBMC cells showed green fluorescence and the other three group had no positive fluorescence.The results indicated that only the 3C1C6G11 hybridoma clones showed anti-PHA-L activity.

In addition, the results in Fig. 1B suggested that monoclonal antibody purified from the ascetic fluid was successfully obtained (the original gel described in Additional file 1). Isotype analysis of monoclonal antibody revealed that mAb 3C1C6G11 was found to belong to the IgG1 subclass and had a kappa light chain.

### Analysis of mAb 3C1C6G11 by western blot

**Fig. 2:**
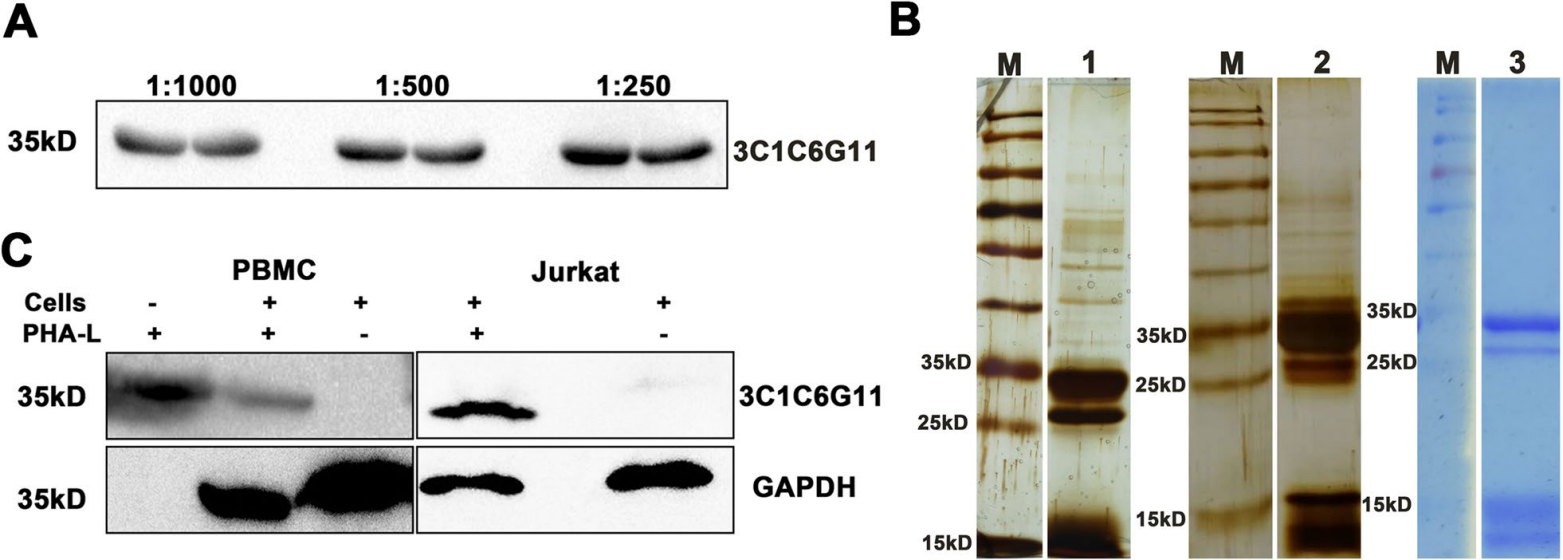
Identification of the specificity of the antibody against the active component of PHA-L. **A** 3C1C6G11 with different dilutions recognizes PHA-L; **B** Protein profile of PHA-L by SDS-PAGE under standard condition. (1. PHA-L from Sigma by SDS-PAGE gel was stained with silver staining; 2. PHA-L from Guangzhou yuanyan biotechnology by SDS-PAGE gel was stained with silver staining; 3. PHA-L from Sigma by SDS-PAGE gel was stained with Coomassie brilliant blue; M. Protein ladder); **C** 3C1C6G11 specifically recognizes PHA-L bound to the human mononuclear cells and Jurkat cells

Western blot (WB) results demonstrated that the antibody 3C1C6G11 could specifically bind to PHA-L at 1:1000, 1:500, 1:250 dilutions, and the recognized band molecular weight was about 35 kD, close to the molecular weight of PHA-L monomer (Fig. 2A). However, commercial PHA-L has many heterobands, and the 15 kD, 25 kD and 35 kD positions are especially evident in Fig. 2B. Therefore, in order to determine the band size of the active component of PHA-L recognized by the mAb 3C1C6G11 was about 35 kD, extracted respectively whole cell proteins of human peripheral blood mononuclear cells (PBMC) and Jurkat cells (human acute T lymphoblastic leukemia cells) treated with PHA-L for WB. As shown in Fig. 2C, with PHA-L as positive control, human monocytes and Jurkat cells were incubated with PHA-L, and bands appeared at 35 kD position, suggesting that mAb 3C1C6G11 can specifically recognize the active PHA-L bound to cells (all the original gels and blots described in Additional file 1).

### The applications of the mAb 3C1C6G11 in immunofluorescence and immunohistochemistry assays

**Fig. 3:**
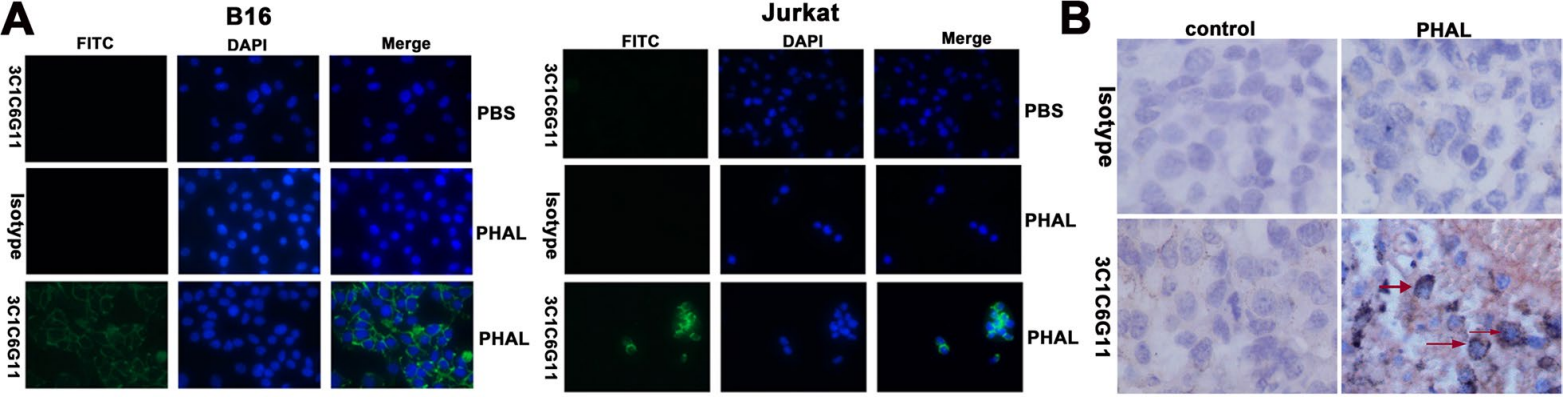
The results of immunofluorescence and immunohistochemistry staining in cells or tissues using mAb 3C1C6G11. **A** The results of immunofluorescence staining in B16 and Jurkat cells incubated with PHA-L or PBS using mAb 3C1C6G11 (original magnification × 400); **B** The results of immunohistochemistry staining in the tumor tissues treated with PHA-L or PBS using mAb 3C1C6G11. The positions of the target cells are indicated by arrows (original magnification × 400)

Immunofluorescence (IF) and immunohistochemistry (IHC) assays were performed to examine whether mAb 3C1C6G11 could be used to recognize the active-PHA-L binding to cells or tissues. Figure 3A demonstrated that the mAb 3C1C6G11 was able to recognize PHA-L binding to cells of different species. The mAb 3C1C6G11 could also identify tumor tissues treated with PHA-L, as demonstrated in Fig. 3B. As a result, the mAb 3C1C6G11 might be applied in immunofluorescence and immunohistochemistry assays.

### The functional activity of the mAb 3C1C6G11

**Fig. 4:**
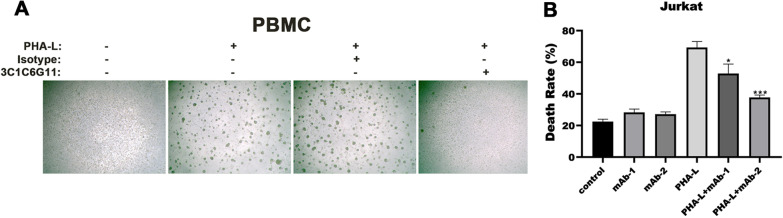
The function of the mAb 3C1C6G11. **A** Representative pictures of blocking PHA-L hemagglutination to human mononuclear cells by the mAb 3C1C6G11 (original magnification × 40); **B** The death rate of Jurkat cells treated with PHA-L or mAb 3C1C6G11 detected by LDH (mAb-1, 1:1000; mAb-2, 1:500)

PHA-L has mitogenic activity and can promote the agglutination of human peripheral blood mononuclear cells (PBMC). By adding antibodies and PHA-L into human monocytes, we found in Fig. 4A that after adding PHA-L, PBMC cells agglutinated considerably, whereas the mAb 3C1C6G11 prevented the agglutination of PHA-L on PBMC cells compared to homologous IgG negative control. The result indicated that the mAb 3C1C6G11 can block the binding of PHA-L to the PBMC cells, making the PHA-L agglutination function weak or disappear.

AICD, activation induced cell death, eliminates autoreactive T or B cells by binding FasL (CD95L) and Fas (CD95) between lymphocytes. Fas-FasL binding induces the transmission of death signal, which is one of the mechanisms of CTL and NK cell killing; however, it can also kill activated lymphocytes and down-regulate cellular and humoral immunity [14]. As demonstrated in Fig. 4B, PHA-L can induce AICD, and we found that the mAb 3C1C6G11 could block PHA-L-induced cell death.

Taken together, these results confirmed once again that the hybridoma clones 3C1C6G11 secreted monoclonal antibody against PHA-L active ingredient.

## Discussion

PHA-L is well known for its promising biomedical applications, especially in the diagnosis and treatment of tumors, while no commercial monoclonal antibodies have been reported for PHA-L research. Our study reported that commercial PHA-L contains high levels of impurities or toxic components. Thus, the preparation of anti-active PHA-L is very necessary to study the function and medicinal value of PHA-L. In this study, commercial PHA-L was employed as an antigen to prepare monoclonal antibodies against the active components of PHA-L. Monoclonal antibody 3C1C6G11 was successfully applied to Western blot, immunohistochemistry and immunofluorescence. Most importantly, the mAb 3C1C6G11 can be used to prevent the detrimental effects of PHA-L, such as cell aggregation and AICD.

As a class of non-immune sugar-binding proteins, plant lectins have been considered by most biomedical scientists for using in therapy a variety of diseases, especially in the field of cancer [15–18]. However, the composition of PHA-L is not single because the general method of preparing lectin involves salt or acid precipitation followed by a variety of chromatographic purification strategies [11–13]. There is an urgent need to evaluate the functional as well as the toxic components of plant lectins to assist in the rational designs of lectin therapy. According to our previous study, PHA-L prepared from crude extracts of red kidney bean by standard strategies, which was a mixture of numerous components. The low content of active ingredients is the reason for its weakening function, and the impurities may also have toxic effects, which is also the main reason for limiting its clinical application [10].

In order to identify and purify the functional components of PHA-L, we prepared antibodies against the active PHA-L. Firstly, four hybridoma clones against full-PHA-L protein were obtained using commercial PHA-L as antigen. Then, anti-active PHA-L mAb named 3C1C6G11 was screened from four hybridoma strains according to the characteristics of PHA-L active ingredient that can bind T lymphocytes, and the monoclonal antibody 3C1C6G11 was purified. WB was used to further verify that the monoclonal antibody 3C1C6G11 only recognized the active components of PHA-L, and the antibody only recognized the bands with molecular weight of about 35 kD, the same as the bands of PHA-L monomer, but could not recognize bands with molecular weight of about 15 kD or 25 kD. These results demonstrate the preparation of a novel monoclonal antibody against the active component of PHA-L. In addition, we performed immunofluorescence and immunohistochemistry on mAb 3C1C6G11 and found that mAb 3C1C6G11 could recognize any PHA-L-bound cells and PHA-L-treated tissues.

In conclusion, the mAb 3C1C6G11 against active-PHA-L can be applied to WB, IF and IHC. As the antibody can bind the active fragment of PHA-L bound to the cell membrane, we can use the antibody to recognize the functional fragment sequence of PHA-L, providing a good identification tool for the application of PHA-L as a drug in clinical therapy. In addition, the antibody can be used to identify the activity of PHA-L, which also provides a simpler and more effective identification method for many researchers in an effort to recombinant express active PHA-L.

Finally, we preliminarily found that the mAb 3C1C6G11 can block cell agglutination and AICD. As we know, hemagglutinating activity of PHA-L is an obstacle to its application in clinical treatment [9, 19–21]. If we can prevent the agglutination of PHA-L and extract its functional components by using mAb 3C1C6G11, it may be an improvement for its application in clinical treatment. AICD can kill activated lymphocytes, which may be one of the mechanisms of tumor immune escape [14]. Thus, if the mAb 3C1C6G11 against active-PHA-L prepared by us can block the occurrence of AICD, it may enhance the activity of immune cells and can be used in anti-tumor treatment. Of course, more research is needed to support our speculation.

In this study, a novel monoclonal antibody against active PHA-L was prepared for the first time. As mentioned above, the anti-active PHA-L monoclonal antibody 3C1C6G11 was obtained by immunization of the full PHA-L protein, which can be used as an important tool to identify the functional sequences of PHA-L and investigate the application value of PHA-L.

## Conclusions

In the present study, one strain of the monoclonal antibodies against active PHA-L named 3C1C6G11 was successfully obtained, and proved that the monoclonal antibody was suitable for WB, IF and IHC, especially with functional activity.

## Methods

### Animals

The female BALB/c mice were purchased from Slake Jingda experimental animal Co., Ltd, Hunan Province, China. All procedures involving animals and their care were conducted in accordance with the guidelines of Medical Ethics Committee of Sichuan University. All the mice mentioned in the present study were euthanized by the method of spine dislocation.

### Reagents

PHA-L (L2769) was purchased from Sigma, Saint Louis, America. PAGE Gel Silver Staining Kit (G7210) was purchased from Solarbio, Beijing, China. CytoTox 96 Non-radioactive Cytotoxicity Assay (G1782) was from Promega, Madison, America. Protein ladder was purchased from Thermo (26616), Lithuania, America. DAB substrate kit was from ZSBIO (ZLI-9017), Beijing, China, and hematoxylin was from Solarbio (G4070). The antibody against GAPDH was purchased from ZEN BIO, ChengDu, China. Human lymphocyte separation solution was purchased from Tianjin Haoyang Biotechnology (Tianjin, China); CD3 monoclonal antibody OKT3 were purchased from CytoCares (Shanghai, China); IL-2 and IFN-γ were purchased from Beijing Tongli Biotechnology (Bejing, China);

### Cell culture

The Jurkat (human acute T cell leukemia cells) and B16-F10 (mouse melanoma cells) cell lines were stored in the Department of immunology, Sichuan University. The cells were maintained in Dulbecco’s Modified Eagleʼs medium or Roswell Park Memorial Institute 1640 medium (Hyclone) supplemented with 10% fetal bovine serum (MHBio, Gansu, China) and cultured at 37 °C in a humidified atmosphere containing 5% CO_2_.

### Isolation of human peripheral blood mononuclear cells

The study was conducted in accordance with the 2013 Declaration of Helsinki. The informed consent was taken from all the volunteers. Peripheral blood samples were collected from 10 healthy volunteers. Among them, 3 were males and 7 were females, ranging in age from 22 to 55 years, with a median age of 29 years.

20 mL anticoagulant was prepared and centrifuged at 1000 g for 15 min. The upper layer of plasma after centrifugation was carefully collected, which can be used as a nutrient component of the culture medium. The precipitate of lower blood cells was slowly added into Ficoll paque and centrifuged at 650 g for 20 min. The middle layer of lymphocytes after centrifugation was taken into the tube and washed with saline for 3 times. The culture medium of T lymphocytes in vitro needs to add a certain concentration of 300 U/mL interleukin-2, 50 ng/mL anti-CD3, and 1,000 U/mL human rIFN-γ, which called “Conditioned medium”.

### Preparation and characterization of monoclonal antibodies

The preparation process of monoclonal antibody includes mouse immunity, cell fusion, hybridoma cell screening and subcloning, and the production and purification of monoclonal antibody. The commercial PHA-L was used as an antigen to produce monoclonal antibodies, seven female 6 to 8-week-old BALB/c mice were injected at multiple sites subcutaneously and intraperitoneally with 100 μg PHA-L protein, thoroughly emulsified with an equal volume of complete Freund’s adjuvant (Sigma). Two boosts were given at days 14 and 28 with 100 μg PHA-L protein thoroughly emulsified with incomplete Freund’s adjuvant (Sigma). Three days after the last boost with 150 μg of the PHA-L protein, the splenocytes of the immunized BALB/c mice were fused with SP2/0 myeloma cells using polyethylene glycol 1500 (PEG 4000) (Solarbio). The hybridomas were selected in RPMI 1640 medium supplemented with hypoxanthine, aminopterin and thymidine (HAT) (Sigma). Positive clones were identified by indirect enzyme linked immunosorbent assay (iELISA), and the steps are as follows: coating antigen PHA-L—blocking—incubation with fusion cell supernatant—washing—incubation with secondary antibody—washing—color development—termination. The results of iELISA showed that the OD value of PHA-L group was high, while the OD value of BSA control group was low, indicating that the cell supernatant was a positive clone, and the secreted antibody had a specific reaction to PHA-L. Then, continue subcloning, and screen until the positive clone is a monoclone (subcloning four times in this experiment). Finally, four hybridoma cell lines 3C1B7A4, 3C1C6G11, 2B7B2A9 and 1G1C4B5 secreting anti PHA-L monoclonal antibodies were established.

Since PHA-L active ingredient can bind to T lymphocytes, we prepared the PBMC; After incubation with 10 μg/mL PHA-L for 4 h, the cells were washed twice with precooled PBS and fixed in 96 well plates. Then add the hybridoma cell line culture supernatant identified as positive by the above indirect ELISA method and subcloned, and conduct immunofluorescence detection. Hybridoma cell lines with anti-PHA-L active components bound to cells were determined. Mice ascites fluids were generated by injecting the hybridomas into the paraffine primed BALB/c mice. After purification by the classical method—Octanoic acid-ammonium sulfate precipitation and Protein G chromatography column, ascites fluid was used for mAb isotyping using an IsoStrip™ Mouse Monoclonal Antibody Isotyping Kit according to the manufacturer’s instructions (this was completed by Changzhou Zhongmei Xinxin biology Co., Ltd).

### SDS-PAGE assays

PHA-L was subjected to SDS-PAGE under two conditions: standard SDS-PAGE and modified SDS-PAGE. For SDS-PAGE under standard condition, the sample was prepared by adding 20 μg of PHA-L into loading buffer with β-mercaptoethanol followed by heating. For SDS-PAGE under modified condition, the sample was prepared by adding equal amount of PHA-L into loading buffer without β-mercaptoethanol and heating. The samples were resolved by 10% SDS-PAGE. Then the gel was subjected to coomassie brilliant blue R-250 staining or silver staining to visualize protein bands in the gel.

### Application of the monoclonal antibodies

Ascites fluid mentioned above was used as the source of murine monoclonal antibodies. By using Goat anti-Mouse IgG Antibody HRP conjugate (ZSBIO) as the second antibody, the mAbs were used for western blot analysis by detecting the active PHA-L bound to Jurkat or PBMC. By using Goat Anti-Mouse IgG with FITC (H + L, ZSBIO) as the secondary antibodies, the mAbs were used for immunofluorescence analysis by detecting the active PHA-L bound to Jurkat and B16 cells. By using SP kit detection system (ZSGB-BIO), the mAbs was used for immunohistochemistry analysis by detecting the active PHA-L bound to B16 tumor tissue.

### Lactate dehydrogenase assay (LDH)

The CytoTox 96® Assay indirectly measures lactate dehydrogenase activity present in the cytoplasm of intact cells. According to the manufactures, cell samples are lysed by adding 15 μL of Lysis 10X Solution per 100 μL of culture medium, followed by incubation at 37 °C for 45–60 min. Sample supernatants (50 μL) are then transferred to a fresh 96-well enzymatic assay plate. CytoTox 96® Reagent (50 μL) is added to each supernatant sample, and the enzymatic reaction is allowed to proceed for 30 min at room temperature, protected from light. The enzymatic assay is then stopped by adding 50 μL/well of Stop Solution. Absorbance can be read at 490 nm using a plate reader. The number of cells present will be directly proportional to the absorbance values, which represent LDH activity.

### Statistical analysis

The data in this paper are expressed as mean ± standard deviation. Graph-Pad Prism 7 statistical software (GraphPad Software Inc., San Diego, CA, USA) was used for single-factor *t*-test analysis. *P* ≤ 0.05 was considered statistically significant.

## Supplementary Information

**Additional file 1**. Original image description of all the gels or immunoblots involved in the article.

## Data Availability

All data generated or analyzed during this study are included in this published article.

## Abbreviations

PHA: Phytohemagglutinin
PHA-L: Leukocyte phytohemagglutinin
mAb: Monoclonal antibody
AICD: Activation-induced cell death
PBMC: Peripheral blood mononuclear cell
WB: Western blot
IF: Immunofluorescence
IHC: Immunohistochemistry
IL-2: Interleukin-2
LDH: Lactate dehydrogenase assay
