# Supplementary material for: Preparation of a novel monoclonal antibody against active components of PHA-L from Phaseolus vulgaris and its functional characteristics
Source: BMC Biotechnol. 2022 Oct 29;22:32. doi: 10.1186/s12896-022-00761-7 (PMC9618193; doi:10.1186/s12896-022-00761-7)

**Supplementary Information**

Figure S1-B. Silver staining analysis of purified mAb 3C1C6G11

Line 1, unpurified ascites fluid; Line 2, ascites fluid after chromatography; Lane 3, purified ascites fluid; M, Protein Ladder.


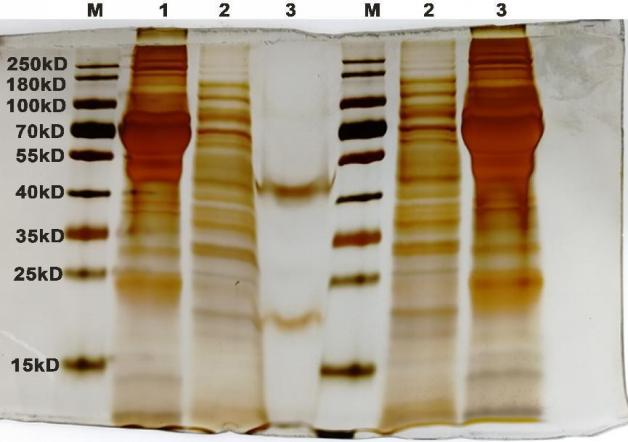


Figure S2-A. 3C1C6G11 with different dilutions recognizes PHA-L

From left to right, the dilution ratio of mAb 3C1C6G11was 1:2000, 1:1000, 1:500 and 1:250.


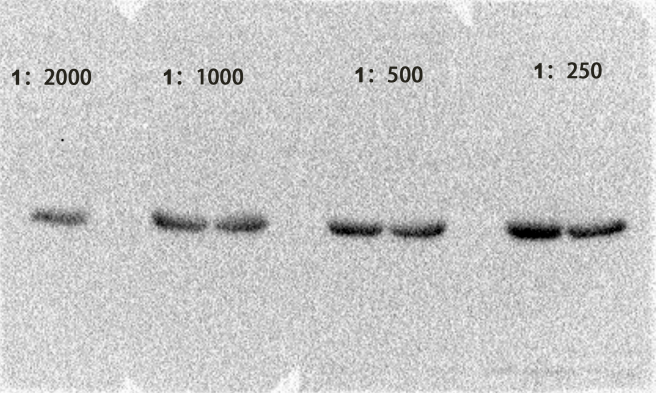


Figure S2-B. PHA-L from Sigma by SDS-PAGE gel was stained with silver staining

Line 1,2 PHA-L protein from Sigma in standard condition; Line 3,4 PHA-L protein from Sigma in modified conditions; M, Protein Ladder.


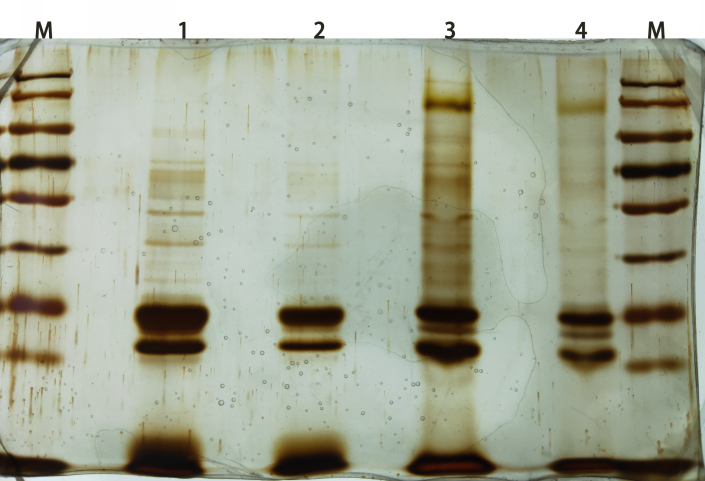


Figure S2-B. PHA-L from Guangzhou yuanyan biotechnology by SDS-PAGE gel was stained with silver staining

Line 1, PHA-L protein from Guangzhou yuanyan biotechnology in modified SDS-PAGE condition; Line 2, PHA-L protein from Guangzhou yuanyan biotechnology in standard SDS-PAGE conditions.


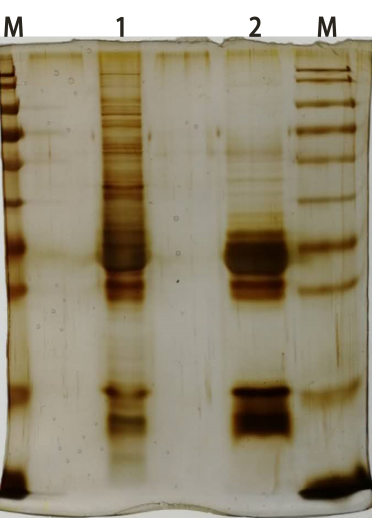


Figure S2-B. PHA-L from Sigma by SDS-PAGE gel was stained with silver staining

Line 1,3 Full PHA-L from Sigma; Line 2,4 Fragment from PHAL; M, Protein Ladder.


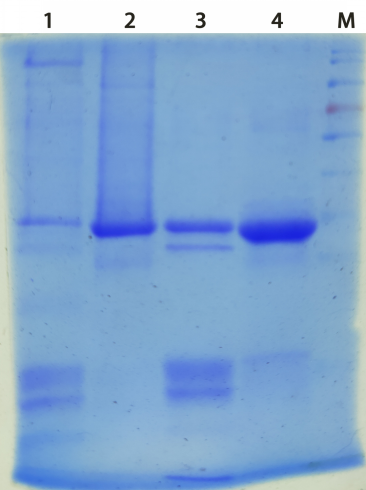


Figure S2-C. 3C1C6G11 specifically recognizes PHA-L bound to the human mononuclear cells.

Line 1, PHA-L protein; Line 2, PBMC with PHA-L; Line 3, PBMC; M, Protein Ladder.


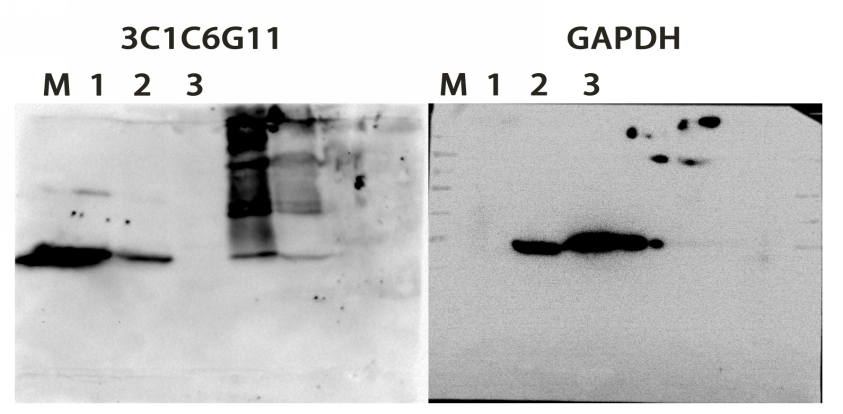


Figure S2-C. 3C1C6G11 specifically recognizes PHA-L bound to the Jurkat cells

Line 1, Jurkat cell with PHAL; Line 2, Jurkat cell; M, Protein Ladder.


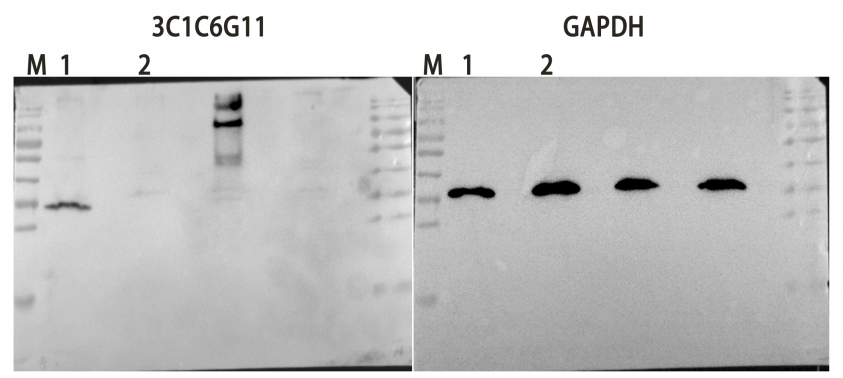

Supplement: Supplementary file 1 — Additional file 1. Original image description of all the gels or immunoblots involved in the article. [file 12896_2022_761_MOESM1_ESM.docx]
